# Genetic Aspects of Problematic and Risky Internet Use in Young Men—Analysis of ANKK1, DRD2 and NTRK3 Gene Polymorphism

**DOI:** 10.3390/genes15020169

**Published:** 2024-01-27

**Authors:** Aleksandra Rył, Natalia Tomska, Anna Jakubowska, Alicja Ogrodniczak, Joanna Palma, Iwona Rotter

**Affiliations:** 1 Department of Medical Rehabilitation and Clinical Physiotherapy, Pomeranian Medical University, 71-210 Szczecin, Poland; natalia.tomska@pum.edu.pl (N.T.); iwona.rotter@pum.edu.pl (I.R.); 2 Department of Genetics and Pathology, Pomeranian Medical University, 71-252 Szczecin, Poland; anna.jakubowska@pum.edu.pl (A.J.); alicja.ogrodniczak@pum.edu.pl (A.O.); 3 Independent Laboratory of Molecular Biology and Genetic Diagnostics, Pomeranian Medical University, 71-252 Szczecin, Poland; 4 Department of Biochemical Science, Pomeranian Medical University, 71-460 Szczecin, Poland; joanna.palma@pum.edu.pl

**Keywords:** gene polymorphism, internet addiction, hormone levels

## Abstract

Background: Internet addiction disorder (IAD) is characterized by an excess of uncontrolled preoccupations, urges, or behaviors related to computer use and Internet access that culminate in negative outcomes or individual distress. PIU includes excessive online activities (such as video gaming, social media use, streaming, pornography viewing, and shopping). The aim of this study was to analyze the association of gene polymorphisms that may influence the severity of risky behaviors in young men with the frequency of Internet use. We speculate that there are individual differences in the mechanisms of Internet addiction and that gene–hormone associations may represent useful biomarkers for subgroups of individuals. Materials and Methods: The study was conducted in a sample of 407 adult males. Subjects were asked to complete the Problematic Internet Use Test (PIUT). Serum was analyzed to determine concentrations of luteinizing hormone (LH), follicle stimulating hormone (FSH), testosterone (TT), sex hormone binding protein (SHBG), dehydroepiandrosterone sulfate (DHEA-S), estradiol (E2), prolactin (PRL), insulin (I), serotonin (5-HT), and dopamine (DA), as well as DRD2, ANKK1, and NTRK3 gene polymorphisms. Results: In the analysis of the ANKK1 gene, there was a specific association between ANKK1 polymorphisms and PRL and 5-HT blood concentrations. There was also an association between the ANKK1 polymorphisms and LH and DA concentrations. When analyzing the DRD2 gene polymorphism, we found that in the group with a moderate level of Internet dependence, there was an association between both the G/GG and GG/GG polymorphisms and FSH concentration. Conclusions: Our study found that there may be an association between the NTRK3 gene polymorphism and PIU. The polymorphisms of ANKK1 and DRD2 genes may be factors that influence the concentrations of hormones (PRL, 5-HT, DA) that are associated with the results obtained in PIU.

## 1. Introduction

Internet addiction disorder (IAD) is a worldwide problem. Studies have shown that IAD is similar to other types of addictive disorders, such as substance dependence, abuse disorders, and pathological gambling [1]. Patients with IAD exhibit clinical features such as eating disorders, withdrawal [2], and increased impulsivity, as well as impaired cognitive performance in decision-making tasks [1]. Despite the magnitude of the problems associated with IAD, not many studies have investigated the genetic basis of IAD. However, a small number of studies have found an association of up to 70% with the inheritance of behavioral addiction [3,4,5,6]. Problematic internet use (PIU) and IAD are similar but not identical problems. PIU includes excessive online activities (such as video gaming, social media use, streaming, pornography viewing, and shopping), while IAD refers to the disorders associated with them. IAD refers to the social, biological, and economic disturbances associated with Internet use [7].

According to the available literature, genes such as DRD2—dopamine receptor D2 gene (rs1799732), ANKK1—ankyrin repeat and kinase domain containing 1 (rs1800497), and NTRK3—neurotrophic receptor tyrosine kinase 3 (rs2229910) influence the development of risky behaviors [8,9]. However, they have been studied in the Asian population and there are no well-documented studies in the European population. 

One of the neurotransmitters long associated with addiction and the rewarding effects of psychoactive drugs is dopamine (DA). It is well known that addictive drugs increase DA release, but current knowledge suggests that the problem is more complex and cannot be limited to dopaminergic activation. It has been found that the C957T polymorphism (SNP: rs1799732), located in exon 7 of DRD2 (gene map locus 11q23), alters the availability of DRD2 and that a higher frequency of the CC genotype is associated with a higher risk of alcoholism [10,11], nicotine dependence [12], and cannabis dependence [13]. In addition, other studies have found that the NTRK3 rs2229910 gene is a genetic variant that is significantly associated with online gaming disorder [14]. It has also been found that polymorphisms in the DRD2/ANKK1 Taq1A-rs1800497 and DAT1-40 pz VNTR genes may be associated with cognitive flexibility (the brain’s ability to adapt human behavior) in people who exhibit behaviors associated with pathological gambling. Pathological gamblers with a genetic predisposition to lower DA and D2 receptor density were more likely to have problems with cognitive flexibility. 

The search for genetic and epigenetic factors seems important from the perspective of studying individual predispositions to PIU. This type of research provides an opportunity to look for molecular factors that may be at the root of the problem.

Another factor that influences human behavior is the concentration of hormones, in particular DA and serotonin (5-HT), hormones that are important in regulating brain processes responsible for controlling human motivation and desire. These are not the only hormones that affect behavior. In men, the concentrations of testosterone (TT), estradiol (E2), and other androgens are also particularly important. Therefore, the following research hypotheses were formulated in the study:

1. The polymorphism of the ANKK1, DRD2 and NTRK3 genes influences the severity of symptoms of Internet addiction in men.
2. The polymorphism of the ANKK1, DRD2 and NTRK3 genes affects the concentration of hormones that regulate men’s behavior.

The aim of the study was to analyze the associations of gene polymorphisms that may influence the severity of risky behaviors in young men with the frequency of Internet use. We investigated three candidate genes that could potentially influence the behavioral phenotype: DRD2—dopamine receptor D2 (rs1799732), ANKK1—ankyrin repeat and kinase domain containing 1 (rs1800497), and NTRK3—neurotrophic tyrosine receptor kinase 3 (rs2229910). They were analyzed for the incidence of PIU and for levels of hormones that affect human behavior.

## 2. Materials and Methods

### 2.1. Study Population

The study was conducted among a sample of 407 adults, male Internet users aged 18 to 30 years (Me = 24.0), living in West Pomerania, NW Poland. Participation in the survey was voluntary. All subjects gave their informed, voluntary, and written consent to participate in the study. The minimum sample size calculated for this study was 384 subjects [15]. 

The study complied with all applicable institutional guidelines for the ethical use of human subjects in research and the tenets of the Declaration of Helsinki. The Ethics Committee of the Medical University of Pomerania approved the study protocol, and all participants gave written informed consent. The material collected from the study participants was analyzed in accordance with the principles of anonymity.

The subjects were asked to fill in our questionnaire (see Supplementary Materials), which included anthropometric data and the Problematic Internet Use Test (PIUT—modified adaptation of the Internet Addiction Test by K. Young, developed by R. Poprawa in 2011, Institute of Psychology at the University of Wrocław) [16]. This questionnaire contains 23 questions about the use of the Internet. The respondent chooses only one answer that best reflects the truth about how often he/she behaves, thinks, or feels in the described way in connection with Internet use. The respondent can answer the question from “never” to “always”. The test can reveal excessive psychological, social, and health problems caused by Internet use—especially with regard to interactivity—that have clear characteristics of addictive behavior. The total raw score is the sum of 23 ratings on a scale from 0 (never) to 5 (always). Minimum raw score = 0, maximum = 110 points. The cut-off points for the PIUT were 0–1 points (very low PIUT); 2–10 (low PIUT); 11–49 average (PIUT); 50–79 (high PIUT); and 80–110 (very high PIUT). The total raw score is converted to tens. The higher the score, the more problematic the use of the Internet.

### 2.2. Test Material Collection

Blood collected from 407 men was drawn from the ulnar vein of the fasted subjects between 7 and 10 am. It was collected into two tubes—3 mL for genetic testing and 9 mL for coagulation to study hormonal parameters. The collected material was centrifuged and the serum and whole blood were stored at −20 °C for further testing. In addition, the patients’ blood pressure, waist circumference, weight and height were measured. Based on these parameters, the body mass index was calculated using the formula BMI = body weight/(height)^2^.

### 2.3. Determination of Biochemical and Hormonal Parameters

Serum concentrations of luteinizing hormone (LH), follicle stimulating hormone (FSH), total testosterone (TT), sex hormone binding protein (SHBG), dehydroepiandrosterone sulfate (DHEA-S), estradiol (E2), prolactin (PRL), and insulin (I) were determined using the electrochemiluminescence immunoassay (ECLIA). The concentration of hormones determined by the ECLIA method [17] was assessed in the certified laboratory Synevo (Poland) which has implemented 3 ISO standards (PN-EN ISO 15189:2013, PN-EN ISO 9001:2015, and PN-ISO/IE 27001:2013). 

Serum concentrations of 5-HT and DA were determined by the ELISA method [18] (Qayee Biotechnology, Shanghai, China). The concentration of hormones determined by the ELISA method was assessed in the laboratory of the Pomeranian Medical University in Szczecin. The method for determining the concentration of 5-HT and DA included the following instruments and steps. Empty wells: without samples and horseradish peroxidase (HRP). Standard wells: 50 μL standard in standard wells. Specimen wells: 40 μL diluent followed by 10 μL specimen. A total of 50 μL horseradish peroxidase (HRP) was added to each well except the blank well. The wells were then gently vortexed and incubated at 37 °C for 60 minutes. Excess fluid was discarded and dried, and each well was filled with diluted cleaning fluid, mixed for 30 s, and the fluid was decanted. This was repeated five times, followed by dry blotting. Incubation was performed for 10 minutes at 37 °C in the dark. In total, 50 μL stop solution was added to each well to stop the reaction. In the final measurement, the blank well was zeroed and the optical density (OD) was measured at a wavelength of 450 nm. According to the concentration of the standards and the corresponding OD values, the linear regression equation of the standard curve was calculated, and then the sample OD values were applied to the regression equation to calculate the concentration of the corresponding sample.

### 2.4. Sample Preparation

To isolate DNA for molecular testing, peripheral blood was collected from each patient to a tube containing EDTA. DNA was isolated from peripheral blood leukocytes using the detergent method [19]. Isolated DNA was stored at 4 °C prior to genotyping.

### 2.5. Genotyping

Molecular analysis was performed using the polymerase chain reaction with real-time PCR analysis using TaqMan hydrolysis probes. The genotyping of 3 tested variations in 3 genes (DRD2, ANKK1, and NTRK3) was performed in a volume of 5 µL, of which 4 µL was the reaction mixture and 1 µL was DNA. Mixtures composed of 2.5 µL GoTaq^®^Probe qPCR Master Mix (Promega, Madison, WI, USA), 0.125 µL TaqMan Genotyping Assays × 40 (Applied Biosystems, Foster City, CA, USA), and 1.375 µL nuclease-free deionized water (Promega, Madison, WI, USA) were used to prepare the reaction mixture. The samples were analyzed on 384-well plates by a LightCycler 480 Multiwell Plate 384 (Roche Diagnostics, North Ryde, Australia) using the LightCycler 480 Instrument thermocycler and the LightCycler 480 Basic Software Version 1.5 computer program (Roche Diagnostics). The fluorescence level of the samples was measured after each reaction cycle. The measurement of the fluorescence intensity of the reporter dyes (FAM and VIC) allowed us to perform the endpoint analysis of the amplification reaction products. The nucleotide sequences of the probes and the types of polymorphisms are as follows: for DRD2 (rs1799732; G/-, GTACCTCCTCGGCGATCCCCGGCCT [G/-] GAACGGGTAGGAGGGGTTGGGGGAT; Reagent catalog number: C_33641686_10, Thermo Fisher Scientific); for ANKK1 (rs1800497, A/G, CACAGCCATCCTCAAAGTGCTGGTC [A/G] AGGCAGGCGCCCAGCTGGACGTCCA; Reagent catalog number: C_7486676_10, Thermo Fisher Scientific); and for NTRK3 (rs2229910, C/G, TGCCAATGACCACAGTGTCGGGCCC [C/G] GCATCCAGTGACGAGGGCGTGGTGA; Reagent catalog number: C_15884895_10, Thermo Fisher Scientific). Real-time PCR reaction parameters are presented in Table 1.

### 2.6. Statistical Analysis

Statistical analysis was performed using Statistica 13.1 (StatSoft Poland, Krakow, Poland). As the distribution of continuous variables deviated from normality, the data are presented as medians and interquartile ranges. Genotype frequencies were tested for the Hardy–Weinberg (H-W) equilibrium using the χ2 test. Statistical inference was based on either the Mann–Whitney U test or the Kruskal–Wallis test. The Chi-square/Fisher’s exact method was used for qualitative data. In the logistic regression analysis, the groups were combined into a group of men with a low level of Internet addiction and men with moderate and high levels of Internet addiction. The significance level was set at *p* < 0.05. For the tests under study, the Bonferroni correction used to correct for multiple comparisons. For each of the loci, we performed analyses in the available inheritance models: DRD2: G/G vs. G/GG GG/GG, G/GG vs. G/G GG/GG, GG/GG vs. G/G G/GG; ANKK1: AA vs. GG AG, AG vs. AA GG, GG vs. AA AG; NTRK3: CC vs. GG CG, CG vs. CC GG, GG vs. CC CG [20].

## 3. Results

### 3.1. Characteristics of the Study Group

In the study group of 407 men between the ages of 18 and 30 (Me = 24.0), 67 subjects showed a low level of Internet addiction, 246 showed a moderate level, and 76 men showed a high level of addiction. Very high problematic Internet use was shown for 3 of the subjects, so they were included in the high-use group for the purpose of data analysis. The most common devices used to access the Internet were “mobile phone and tablet” (54%), followed by computer (29%) and mobile phone only (17%). Respondents used the Internet more than 3 h a day for work or study and more than 4 h a day for social networking.

### 3.2. Genotyping Results

Each locus was studied in accordance with the Hardy–Weinberg principle (DRD2: rs1799732, *p* = 0.781; ANKK1: rs1800497, *p* = 0.472; NTRK3: rs2229910, *p* = 0.697). We found differences in the degree of Internet addiction between the following polymorphic variants of the NTRK3 gene: GG + CG vs. CC (*p* = 0.007) and GG + CC vs. CG (*p* = 0.017). No differences were found for the variants of other genes (Table 2).

We analyzed the differences in the concentration of hormones and in the problematic Internet use between the selected gene polymorphisms (Table 3). All three groups of ANKK1 gene polymorphism differed in PRL concentration (*p* = 0.032). An analysis between a single polymorphism and groups of polymorphisms also showed a difference in PRL levels for AA vs. GG AG (*p* = 0.013) and AG vs. AA GG (*p* = 0.018).

When analyzing the differences in the concentration of hormones that regulate human behavior between selected DRD2 gene polymorphisms, we found differences in FSH levels for G/GG vs. G/G + GG/GG (*p* = 0.021) and GG/GG vs. G/G + G/GG (*p* = 0.028). Differences in E2 levels were also found for G/GG vs. G/G + GG/GG (*p* = 0.033). When analyzing the NTRK3 gene polymorphism, we found differences in DHEA-S for CG vs. CC + GG (*p* = 0.001) and for GG vs. CC + CG (*p* = 0.013), as well as in PRL concentrations for CC vs. GG + CG (*p* = 0.018).

The differences in the concentrations of hormones between gene polymorphisms were also analyzed depending on the degree of Internet addiction. Table 4 shows only statistically significant differences. In the group of men with a moderate level of Internet addiction, in the analysis of the ANKK1 gene, the GA polymorphism differed from the other polymorphisms in the levels of PRL (*p* = 0.009) and 5-HT (*p* = 0.043). We also found a difference between AA polymorphism and other polymorphisms in LH (*p* = 0.037) and DA levels (*p* = 0.033). 

With regard to the DRD2 gene polymorphism, we found that in the group with a moderate level of Internet dependence, there was a difference in FSH levels between the G/GG polymorphism and two other polymorphisms (*p* = 0.041) and a difference in FSH between GG/GG polymorphism and two other polymorphisms (*p* = 0.026). In men with a high degree of Internet addiction, we found that men with the G/GG polymorphism differed in PRL concentration from those with the two other polymorphisms (*p* = 0.035) and GG/GG differed in PRL from two other polymorphisms (*p* = 0.047). In men with a high degree of dependence, differences in LH (*p* = 0.028) and DHEA-S (*p* = 0.049) were found between CG and the rest of the polymorphisms.

Correlation analysis was also performed between Internet addiction and the levels of hormonal parameters in polymorphic gene clusters. This analysis showed that the level of Internet addiction correlated with the level of SHGB (R = 0.210, *p* = 0.023) in people with the heterozygous genotype AG of the ANKK1 gene. When analyzing the DRD2 gene polymorphism, we found that in people with the GG/GG polymorphism, the level of Internet addiction correlated with the concentration of E2 (R = 0.12, *p* = 0.034). However, when we analyzed the NTRK3 gene, we found that in people who are heterozygous for CG, the level of Internet addiction correlated with both DHEA-S (R = 0.15, *p* = 0.040) and E2 (R = 0.15, *p* = 0.038). 

A logistic regression analysis (Table 5) was also performed with low, moderate, and high levels of Internet addiction as explanatory variables. We found that in the CG NTRK3 heterozygote, the scores obtained in the Internet addiction questionnaire were negatively related to the concentration of DA (OR = 0.986, *p* = 0.039). 

## 4. Discussion

The search for evidence of a molecular genetic link between serotonergic and dopaminergic neurotransmission and Internet addiction is a new look at the problem of behavioral addiction [21]. In our study, we found that not only can a gene polymorphism indirectly affect the severity of the problem itself but also the levels of hormones that regulate human behavior. When analyzing the direct association between genes and Internet addiction, attention must be paid to the NTRK3 gene, whose polymorphism seems to directly regulate the severity of the PIU problem.

The NTRK3 gene encodes a membrane-associated receptor with a high affinity for neurotrophic factor 3 (NTF3). It is responsible for the process of myelination and apoptosis of neurons and glial cells [22]. Therefore, by affecting the integrity of the white matter, the polymorphism of the NTRK3 gene may be a factor that influences a person’s susceptibility to IAD. Although this type of SNP appears to be a silent SNP, a type of mutation that does not affect the phenotype [14], these mutations can affect the level of gene expression by modulating splicing patterns and mRNA stability [23,24]. 

Different genes may be involved in regulating the onset and development of behavioral addictions. Research conducted by Kim et al. [14] on the neurotrophic tyrosine receptor kinase 3 (NTRK3, rs2229910) found a significant difference in the minor allele frequency in IAD subjects compared to controls (*p* = 0.019). They found that the NTRK3 gene polymorphism may play a protective role and reduce the risk of PIU (OR = 0.154). In addition, the presence of a protective allele was associated with less time spent playing online games and lower scores on Young’s Internet Addiction Test. In another study, Jeong et al. conducted further genetic testing [8] on 83 polymorphic regions in a group of 30 people with IAD and found statistically significant associations of IAD with only one SNP, rs2229910, associated with anxiety, depression, obsessive-compulsive disorder, and nutritional disorders. In our study, we found that there may be an association between NTRK3 genotype and DA levels in groups with different degrees of PIU. 

The biological relationship between dopamine D2 receptor and Internet addiction involves complex neurobiological mechanisms. Dopamine, a neurotransmitter associated with reward and reinforcement, plays a critical role in the development and maintenance of addictive behaviors. The excessive and prolonged stimulation of the brain’s reward system by Internet use may lead to adaptations in the dopamine receptor D2 system. Research suggests that prolonged and heightened Internet-related stimuli may induce changes in dopamine receptor D2 sensitivity. This phenomenon, known as dopamine receptor D2 downregulation, is characterized by a reduction in receptor responsiveness. As a result, individuals may experience decreased sensitivity to natural rewards, contributing to the development of tolerance and the pursuit of more intense stimuli. Alterations in the dopamine system may influence motivational processes, decision-making, and the formation of habitual behaviors associated with excessive Internet use [19]. 

In our study, we analyzed the DRD2/ANKK1 Taq1A polymorphism (rs1800497), the SNP of the dopamine receptor D2 gene that encodes the restriction fragment length polymorphism [25]. This polymorphism has been identified in exon 8 of the ankyrin repeat and kinase domain containing 1 gene (ANKK1), causing a Glu713-na-Lys substitution in the putative ANKK1 protein [26]. It is associated with the differential availability of the dopamine receptor D2 [27] and has sparked interest in genetic research on behavioral addictions. The del+ genotype was associated with a more frequent display of novelty-seeking behaviors, whereas the del- genotype was associated with the display of behaviors related to escaping negative emotions in the IAD group. The study found no direct association between the Taq1A polymorphism and PIU. It was concluded that it may indirectly influence personality traits through an indirect interaction [6,11]. 

ANKK1 polymorphism results in low dopamine receptor D2 expression in the prefrontal cortex and is more common in male adolescents with Internet gaming addiction [10,28]. This polymorphism is also more frequent in gamers with excessive reward-seeking [6,27]. Our study found that the DRD2 /ANKK1 Taq1A polymorphism was not directly associated with more risky Internet use behaviors in young men. However, we found that this polymorphism—particularly the presence of the A allele—may be related to levels of hormones that regulate human behavior. In the analysis, we found that men with a moderate level of PIU with the A allele may have higher levels of PRL, 5-HT, and DA. This relationship was not found in the group of men with low and high levels of this problem.

The search for addictions and the genetic basis of Internet addiction is important because of the growing problem of Internet use. Social background and economic conditions do not seem to fully explain this problem. Men’s hormone levels are one factor that seems to explain a lot of male behavior, especially in relation to risky behavior. As no studies have shown a direct relationship between PIU and hormone levels, our research seems to be groundbreaking in this regard.

There are not many studies on the association between DRD2 gene polymorphism and PIU in the available literature. However, genetic polymorphisms that alter the availability and expression of the dopamine receptor D2 are associated with both substance and behavioral addictions [29]. The DRD2 polymorphism (SNP: rs1799732) C957T is located in exon 7 of DRD2 (locus 11q23). It alters the availability of DRD2, and in the presence of the CC genotype has been associated with alcoholism [11].

The biological mechanism underlying the association between the DRD2 gene polymorphism and Internet addiction involves the dopaminergic system, specifically the D2 subtype of the dopamine receptor D2. The DRD2 gene encodes the D2 receptor, a critical component of the dopaminergic neurotransmission pathway. Polymorphisms in the DRD2 gene can result in variations in the density and function of D2 receptors. 

A common polymorphism associated with addiction is the Taq1A polymorphism located in the ANKK1 gene region. This polymorphism has been associated with altered dopamine receptor D2 density and affinity, which influences an individual’s susceptibility to addictive behaviors, including Internet addiction. Research suggests that certain DRD2 gene polymorphisms may contribute to a hypodopaminergic state in which reduced dopamine receptor availability is associated with reduced reward sensitivity. This diminished reward responsiveness may lead individuals to seek increased stimulation, which may manifest as addictive behaviors, such as compulsive Internet use. Understanding the genetic basis, specifically the DRD2 gene polymorphisms, provides insight into the neurobiological underpinnings of Internet addiction. However, it is important to recognize that genetic factors interact with environmental influences in a complex manner, contributing to the multifaceted nature of Internet addiction etiology [30]. 

In our analysis, we found no association between the polymorphism of DRD2 and PIU. The levels of PRL and 5-HT were dependent on the genetic variant of DRD2. In men with a high degree of PIU, individuals with the GG/GG genotype seemed to have higher PRL levels compared to other genetic variants. This is particularly important when analyzing behavioral problems in men with PIU. Our recent study showed that there is an association between the severity of depression symptoms and the incidence of anxiety, verbal and physical aggression, and problematic Internet use [31]. The significance of that study is that it shows how problematic Internet use can lead to the development of negative emotions such as anxiety, anger, aggression, or despair. Higher PIUT scores were associated with longer periods of Internet engagement. The frequency of anxiety, verbal and physical violence, and problematic Internet use was correlated with the severity of moderate depression symptoms. Given the consequences of long-term elevated prolactin levels in men, such as impaired sexual function, this is an issue that needs further investigation. The consequences of high prolactin levels in men may include disturbances in testicular function, TT production, and decreased sperm production [32]. In the context of PIU, there are associations between hormonal imbalances and addictive behaviors. Some studies suggest that hormonal dysregulation may impair stress coping mechanisms and mood regulation, thereby increasing susceptibility to various addictions, including Internet-related behaviors. High levels of prolactin may disrupt hormonal and neurochemical balance, affecting mood regulation and motivation [32,33,34].

The interest in hormonal changes in individuals with PIU is based on the growing evidence for the involvement of the hypothalamic–pituitary–adrenal axis in both substance-use disorders and behavioral addictions [35]. However, studies mainly examine the concentrations of cortisol, leptin, 5-HT, and DA. There are few studies analyzing the concentration of endorphins in the context of PIU. Koeniga et al. examined the concentration of TT, progesterone, dehydroepiandrosterone (DHEA), and corticosterone, without finding differences between patients with Internet gaming disorder (n = 31) and healthy controls (n = 31) [36]. In males, androgens are important in producing sex-appropriate behavior. However, the relationship between PIU and physical activity limitation and lifestyle modification remains unexplored. In the literature, 2D:4D marker tests have been performed by Müller et al. [37]. This ratio is considered to be a biomarker of the balance between fetal TT and estrogen in a narrow window of early ontogeny. By analyzing the literature, they showed that the 2D:4D marker is an interesting marker for Internet addiction and can be easily incorporated as a biomarker to understand the biological basis of Internet use.

In our study, we have shown that there may be a relationship between DA concentration and PIU in relation to the genes analyzed. The involvement of the dopaminergic neural system in IAD has been investigated in several studies [30,31]. Individuals diagnosed with IAD showed alterations in resting glucose metabolism in several brain regions, particularly in key DA projection areas such as the striatum and orbitofrontal region [38]. In addition, adolescents with elevated genetic polymorphisms in genes responsible for the dopamine D2 receptor and the DA-degrading enzyme showed a greater susceptibility to excessive Internet gaming compared to a control group of the same age [27].

### Limitations of the Study 

Many studies of problematic Internet use employ multiple questionnaires with different cut-offs, making it difficult to provide accurate comparisons between populations, despite the wealth of publications on the subject. In addition, a questionnaire survey is a subjective method in which the respondent’s mood at the time of the survey is only one of many variables that may influence their response. The ambiguity and variety of terms used can make it difficult to detect problematic Internet use. Both research and clinical practice will benefit from the identification of protective and risk variables for Internet addiction. A limitation of the study is the assessment of hormone levels at the peripheral rather than the central level. Also, we relied on a single measurement not depending on the actual Internet use. Another limitation of the study is that it was conducted only in a group of men. Finally, conducting this type of research on both women and men would allow for a better understanding of the issue of Internet addiction.

## 5. Conclusions

Genetic research is a new approach to the problem of behavioral addictions, especially those associated with the development of new technologies. However, the increasing magnitude of the problem of PIU and its impact on health necessitates the search for new genetic and epigenetic factors. Our study found that there may be an association between the NTRK3 gene polymorphism and PIU. The polymorphism of the ANKK1 and DRD2 genes may be a factor that affects the concentration of hormones (PRL, 5-HT, DA) depending on the result obtained in PIU, which may be associated with health consequences for men in their older age.

## Figures and Tables

**Table 1:** Real-time PCR reaction parameters.

Program	Proces	Temperature [°C]	Time	Number of Cycles
Incubation	HotStart	95	10 min	1
Amplification and data collection	Denaturation	95	10 s	40
	Attaching the primers	60	30 s	
	Elongation	72	1 s	
Cooling	Cooling	40	30 s	-
Color compensation	Denaturation	95	1 s	
	Cooling	40	30 s	
	Hybridization	67	All the time (1 reading/°C)	
	Cooling	40	45 s	
Melting curve	Hybridization	60	1 s	
	Hybridization	61	All the time (5 reading/°C)	
Cooling	Cooling	40	-	

**Table 2:** Number of ANKK1, DRD2, NTRK3 gene polymorphisms in groups of men with different degrees of Internet addiction, chi-square test.

	ANKK1			*DRD2*			*NTRK3*		
Internet Addiction	AG + AA	GG	*p*	G/GG + GG/GG	G/G	*p*	GG + CG	CC	*p*
high	23 (5.65%)	44 (10.81%)	0.531	66 (16.18%)	2 (0.49%)	0.295	59 (14.50%)	8 (1.97%)	0.007 *
average	89 (21.87%)	175 (43.00%)		260 (63.73%)	4 (0.98%)		213 (52.33%)	51 (12.53%)	
low	22 (5.41%)	54 (13.27%)		76 (18.63%)	0 (0.00%)		72 (17.69%)	4 (0.98%)	
	GG + AA	AG		G/G + GG/GG	G/GG		GG + CC	CG	
high	45 (11.06%)	22 (5.41%)	0.548	52 (12.78%)	15 (3.69%)	0.483	34 (8.35%)	33 (8.11%)	0.017 *
average	189 (46.44%)	75 (18.43%)		214 (52.58%)	50 (12.29%)		146 (35.87%)	118 (28.99%)	
low	55 (13.51%)	21 (5.16%)		59 (14.50%)	17 (4.18%)		28 (6.88%)	48 (11.79%)	
	GG + GA	AA		G/GG + G/G	GG/GG		CC + CG	GG	
high	66 (16.22%)	1 (0.25%)	0.144	18 (4.41%)	50 (12.25%)	0.443	41 (10.07%)	26 (6.39%)	0.499
average	250 (61.43%)	14 (3.44%)		54 (13.24%)	210 (51.47%)		169 (41.52%)	95 (23.34%)	
low	75 (18.43%)	1 (0.25%)		17 (4.17%)	59 (14.46%)		52 (12.78%)	24 (5.90%)	

A—adenine; C—cytosine; G—guanine; *n*—number; *p*—statistical significance; *—statically significant parameter (*p* < 0.05).

**Table 3:** The association between selected gene polymorphisms and the concentration of hormonal parameters and risky behavior with regard to Internet use.

Gene *ANKK1* Polymorphism Analysis										
Variable	GG = 273		AG, n = 118		AA, n = 16		*p* ^1^	AA vs. GG + AG ^2^	AG vs. AA + GG ^2^	GG vs. AA + AG ^2^
	Me	SD	Me	SD	Me	SD				
PIUT	13.00	17.15	16.00	17.44	17.50	9.67	0.443	0.562	0.548	0.334
LH	5.33	11.77	5.53	2.56	6.33	4.73	0.128	0.134	0.179	0.462
FSH	3.38	13.77	3.66	2.65	4.21	9.09	0.375	0.382	0.631	0.462
TT	4.83	2.23	4.92	2.10	5.05	1.78	0.522	0.412	0.569	0.247
SHGB	29.56	14.73	32.45	13.03	30.21	9.26	0.549	0.112	0.387	0.397
DHEAS	354.25	122.88	376.30	158.67	389.80	99.29	0.106	0.161	0.610	0.534
E2	24.60	9.60	24.70	8.17	27.10	9.57	0.283	0.534	0.562	0.486
PRL	237.50	151.91	214.00	113.68	273.00	83.08	0.032 *	0.013 *	0.018 *	0.612
5-HT	95.49	91.49	106.88	143.81	72.34	124.29	0.167	0.0 44	0.607	0.002
DA	88.72	40.50	79.87	37.82	53.26	17.60	0.059	0.120	0.117	0.183
Gene *DRD2* polymorphism analysis										
Variable	GG/GG, n = 319		G/GG, n = 82		G/G, n = 6		*p* ^1^	G/G vs. G/GG + GG/GG ^2^	G/GG vs. G/G + GG/GG ^2^	GG/GG vs. G/G + G/GG ^2^
	Me	SD	Me	SD	Me	SD				
PIUT	14.00	16.93	14.00	17.26	15.50	21.81	0.559	0.418	0.580	0.518
LH	5.39	10.88	5.52	4.26	4.97	4.22	0.251	0.552	0.373	0.349
FSH	3.58	12.07	3.18	9.68	3.50	1.24	0.069	0.588	0.021 *	0.028 *
TT	4.94	2.24	4.84	1.98	4.58	1.71	0.417	0.389	0.336	0.265
SHGB	30.90	13.92	31.23	14.88	28.79	10.51	0.617	0.628	0.524	0.532
DHEAS	368.20	140.14	338.80	97.40	398.70	221.26	0.244	0.525	0.107	0.140
E2	25.00	9.10	22.70	9.58	29.00	6.99	0.007 *	0.315	0.033 *	0.070
PRL	238.00	121.63	229.00	194.95	225.00	88.12	0.545	0.476	0.466	0.415
5-HT	94.80	93.59	117.84	156.89	78.95	102.63	0.339	0.625	0.164	0.182
DA	81.28	39.31	88.06	41.90	96.19	41.60	0.456	0.425	0.363	0.294
Gene *NTRK3* polymorphism analysis										
Variable	CG, n = 145		GG, n = 199		CC, n = 63		*p* ^1^	CC vs. GG + CG ^2^	CG vs. CC + GG ^2^	GG vs. CC + CG ^2^
	Me	SD	Me	SD	Me	SD				
PIUT	13.00	17.13	15.00	16.78	18.00	17.18	0.147	0.070	0.157	0.613
LH	5.40	9.95	5.53	11.43	4.95	2.19	0.454	0.327	0.596	0.352
FSH	3.33	13.33	3.50	11.25	3.74	2.19	0.357	0.390	0.212	0.460
TT	4.81	2.29	5.09	2.10	4.75	1.96	0.237	0.136	0.628	0.225
SHGB	29.11	14.49	32.16	13.46	28.47	14.09	0.206	0.238	0.375	0.104
DHEAS	373.50	138.62	340.25	130.07	350.60	121.22	0.006 *	0.240	0.001 *	0.013 *
E2	24.15	9.84	24.60	8.85	25.10	8.04	0.634	0.602	0.628	0.600
PRL	235.00	124.64	230.50	122.95	273.00	203.61	0.059	0.018 *	0.271	0.369
5-HT	97.42	107.58	98.80	104.24	93.95	126.50	0.604	0.615	0.497	0.512
DA	71.60	41.89	78.11	40.25	96.19	32.50	0.515	0.365	0.606	0.466

^1^—the relationship between three groups (Kruskal–Wallis test), ^2^—the relationship between two groups (Mann–Whitney U-test), Me—median, SD—standard deviation, n—number, PIUT—Problematic Internet Use Test result, LH—luteinizing hormone, FSH—follicle stimulating hormone, TT—total testosterone, SHBG—sex hormone binding protein, DHEA-S—dehydroepiandrosterone sulfate, E2—estradiol, PRL—prolactin, 5-HT—serotonin, DA—dopamine, *p*—statistical significance; *—statistically significant parameter (*p* < 0.05, after Bonferroni correction).

**Table 4:** The association between gene polymorphisms and the concentration of hormonal parameters was also analyzed in the groups, depending on the degree of Internet addiction.

ANKK1 Gene Polymorphism Analysis									
Variables	Low Internet Addiction			Average Level of Internet Addiction			High Internet Addiction		
	Me (Q1; Q3)	Me (Q1; Q3)	*p*	Me (Q1; Q3)	Me (Q1; Q3)	*p*	Me (Q1; Q3)	Me (Q1; Q3)	*p*
	GA, n = 22	AA + GG, n = 55		GA, n = 75	AA + GG, n = 189		GA, n = 22	AA + GG, n = 45	
PRL	216.50 (171.00; 348.00)	231.00 (172.00; 303.00)	0.517	212.00 (167.00; 283.00)	247.50 (198.00; 329.50)	0.09	216.00 (164.00; 321.00)	242.00 189.00; 354.00)	0.298
5-HT	56.15 (36.21; 112.90)	112.02 (37.23; 166.45)	0.260	124.94 (87.49; 163.91)	96.97 (45.54; 154.41)	0.042	94.56 (57.42 213.31)	91.91 (53.82; 141.44)	0.454
	AA, n = 1	GG + GA, n = 75		AA, n = 14	GG + GA, n = 250		AA, n = 1	GG + GA, n = 66	
LH	4.46 (4.13; 4.96)	5.62 (4.47; 7.41)	-	6.33 (4.95; 8.41)	5.22 (3.82; 6.94)	0.036	9.38 (6.88; 12.27)	6.10 (4.74; 7.19)	-
DA	62.25 (60.21; 65.45)	90.14 (56.63; 130.20)	-	52.79 (44.98; 55.63)	76.59 (53.20; 108.25)	0.033	102.22 (85.75; 120.92)	103.40 (49.25; 117.23)	-
DRD2 gene polymorphism									
	G/GG, n = 17	G/G + GG/GG, n = 59		G/GG, n = 50	G/G + GG/GG, n = 114		G/GG, n = 15	G/G + GG/GG, n = 52	
FSH	3.45 (2.83; 4.21)	4.14 (2.67; 6.07)	0.179	2.76 (2.10; 3.99)	3.50 (2.48; 4.89)	0.040	3.41 (2.53; 5.44)	3.74 (2.76; 4.33)	0.517
PRL	226.00 (167.50; 288.50)	231.00 (172.00; 325.00)	0.586	239.50 (189.50; 349.50)	237.00 (187.00; 303.00)	0426	176.50 (155.00; 295.00)	255.00 (191.00; 358.00)	0.034
	GG/GG, n = 59	G/G + G/GG, n = 17		GG/GG, n = 210	G/G + G/GG, n = 54		GG/GG, n = 50	G/G + G/GG, n = 18	*p*
FSH	4.14 (2.67; 6.07)	3.45 (2.83; 4.21)	0.179	3.52 (2.48; 4.90)	2.76 (2.19; 3.83)	0.026	3.65 (2.68; 4.33)	3.90 (2.74; 5.25)	0.370
PRL	231.00 (172.00; 325.00)	226.00 167.50; 288.50)	0.186	237.00 (187.00; 303.00)	239.50 (189.50; 342.50)	0.446	255.00 (191.00; 358.00)	179.00 (158.00; 299.00)	0.046
NTRK3 gene polymorphism									
	CC, n = 4	GG + CG, n = 72		CC, n = 51	GG + CG, n = 213		CC, n = 8	GG + CG, n = 59	
PRL	158.00 (127.00; 504.00)	231.50 (172.00; 324.00)	0.462	278.00 (224.00; 345.00)	234.00 (178.00; 295.00)	0.007	329.00 (234.50; 374.00)	232.00 (177.00; 354.00)	0.267
	CG, n = 28	GG + CC, n = 48		CG, n = 118	GG + CC, n = 146		CG, n = 33	GG + CC, n = 34	
LH	5.70 (4.58; 7.42)	5.44 (4.44; 7.39)	0.365	5.09 (4.00; 6.49)	5.52 (3.80; 7.46)	0.252	6.54 (5.36; 7.42)	5.44 (4.01; 6.83)	0.028
DHEAS	334.60 (265.70; 403.80)	364.35 (327.70; 448.50)	0.133	380.30 (305.20; 461.70	344.50 (263.10; 443.80	0.039	445.30 (315.60; 551.20	350.80 (284.25; 418.30	0.248
E2	22.70 (17.50; 27.80)	24.00 (19.10; 30.40)	0.410	24.10 (19.90; 32.00)	25.25 (20.50; 31.00)	0.584	28.00 (23.80; 35.00)	24.85 (19.70; 30.75)	0.155
DA	62.75 (49.05; 86.10)	107.20 (68.38; 137.46)	0.02	60.82 (50.08; 96.15)	88.72 (54.38; 110.59)	0.069	104.12 (49.88; 130.65)	102.81 (44.93; 111.41)	0.228

Me—median, SD—standard deviation, Q1—lower quartile, Q3—upper quartile, n—number, PRL—prolactin, 5-HT—serotonin, DA—dopamine, LH—luteinizing hormone, FSH—follicle stimulating hormone, SHBG—sex hormone binding protein, DHEA-S—dehydroepiandrosterone sulfate, E2—estradiol, *p*—statistical significance.

**Table 5:** Logistic regression for single polymorphisms of the NTRK3 gene—neurotrophic tyrosine kinase receptor type 3 (rs2229910). Explanatory variable, low, medium and high Internet addiction.

Effect	CC				CG				GG			
	*p*	OR	Cl −95%	Cl +95%	*p*	OR	Cl −95%	Cl +95%	*p*	OR	Cl −95%	Cl +95%
SHGB	0.909	1.194	0.058	24.746	0.212	1.026	0.986	1.068	0.773	0.994	0.953	1.037
DHEAS	0.995	0.999	0.849	1.177	0.140	1.004	0.999	1.009	0.670	1.001	0.995	1.008
E2	0.731	2.117	0.029	152.387	0.152	1.049	0.983	1.120	0.376	1.047	0.946	1.158
PRL	0.845	0.961	0.646	1.430	0.165	1.003	0.999	1.008	0.729	0.999	0.993	1.005
5-HT	0.888	1.050	0.534	2.065	0.254	1.005	0.997	1.013	0.259	0.997	0.992	1.002
DA	0.994	0.997	0.453	2.196	0.039 *	0.986	0.973	0.999	0.989	1.000	0.984	1.016

*p*—statistical significance, OR—odds ratio, Cl −95%—confidence interval −95%, Cl +95%—confidence interval +95%, SHBG—sex hormone binding protein, DHEA-S—dehydroepiandrosterone sulfate, E2—estradiol, PRL—prolactin, 5-HT—serotonin, DA—dopamine, *—statistically significant parameter (*p* < 0.05).

## Data Availability

The data presented in this study are available on request from the corresponding author.

## Supplementary Materials

The following supporting information can be downloaded at: https://www.mdpi.com/article/10.3390/genes15020169/s1, Questionnaire: Polish version of Problematic Internet Use Test titled in Polish language “Test Problematycznego Używania Internetu”.
