# Supplementary material for: Genetic Aspects of Problematic and Risky Internet Use in Young Men—Analysis of ANKK1, DRD2 and NTRK3 Gene Polymorphism
Source: Genes (Basel). 2024 Jan 27;15(2):169. doi: 10.3390/genes15020169 (PMC10887871; doi:10.3390/genes15020169)
Supplement: Supplementary file 1 [file genes-15-00169-s001.zip › genes-2793504-supplementary.pdf]

Test Problematycznego Używania Internetu

Przeczytaj uważnie każde stwierdzenie dotyczące Twojego korzystania z Internetu. Zaznacz tylko **jedną odpowiedź**, która **najlepiej** oddaje prawdę na temat tego, jak często zachowujesz się, myślisz lub czujesz w opisany sposób, w związku z Twoim używaniem Internetu. Wykorzystuj cały zakres możliwych odpowiedzi, od „nigdy" do „zawsze". Kiedy mowa jest o „przebywaniu online" lub w „sieci", mamy na myśli Twoje korzystanie z Internetu.

	Proszę wskazać znakiem „X" jedną odpowiedź.	nigdy	sporady-cznie	rzadko	czasami	często	zawsze
1.	Uświadamiam sobie, że przebywam w sieci dłużej, niż pierwotnie zamierzałam/em						
2.	Zaniedbuję obowiązki domowe na rzecz dłuższego przebywania online.						
3.	Wybieram podekscytowanie związane z Internetem, zamiast bliskości mojego/mojej partnera/partnerki, przyjaciół lub rodziny.						
4.	Tworzę nowe związki z współużytkownikami sieci kosztem związków z osobami z poza sieci.						
5.	Osoby z mojego otoczenia skarżą się na ilość czasu, który spędzam online.						
6.	Zdarza mi się mówić: „jeszcze tylko kilka minut", gdy jestem w sieci.						
7.	Z powodu zbyt długiego czasu spędzanego w sieci, narażam na szwank swoje wyniki w pracy/szkole.						
8.	Zdarza mi się ukrywać co naprawdę robię w sieci, gdy jestem o to pytana/y.						
9.	Wchodząc do Internetu uspokajam niepokojące myśli dotyczące mojego życia.						
10.	Uświadamiam sobie, że myślę o tym, kiedy znów będę w sieci.						
11.	Obawiam się, że moje życie bez Internetu byłoby nudne, puste i pozbawione radości.						
12.	Tracę panowanie nad sobą lub krzyczę, gdy ktoś przeszkadza mi gdy jestem online.						
13.	Zdarza mi się zaniedbywać sen z powodu długiego przebywania w sieci.						
14.	Czuje się zaabsorbowana/y Internetem gdy jestem offline i fantazjuję na temat bycia online.						
15.	Moje osiągnięcia w pracy/szkole cierpią z powodu zbyt długiego czasu spędzanego przeze mnie w sieci.						
16.	Próbowałam/em bez powodzenia zredukować długość trwania moich sesji w Internecie.						
17.	Próbuję ukrywać przed innymi to, jak długo przebywam w sieci.						
18.	Wybieram spędzanie czasu w sieci zamiast spotykania się z przyjaciółmi lub rodziną.						
19.	Czuję się poirytowana/y, nerwowa/y lub przygnębiona/y gdy jestem offline, ale uczucia te znikają gdy wracam do sieci.						
20.	Bycie online pozwala mi uśmierzyć moje negatywne uczucia (np. beznadziejności, smutku, przygnębienia, lęku czy winy).						
21.	Czuję się niespokojna/y, poirytowana/y czy przygnębiona/y myślą o konieczności ograniczenia korzystania z Internetu.						
22.	Zauważam, że muszę wydłużać czas spędzany w Internecie aby osiągnąć satysfakcję z korzystania z sieci.						

zmodyfikowana adaptacja Internet Addiction Test K.Young w opracowaniu R.Poprawy z 2011, Instytut Psychologii Uniwersytetu Wrocławskiego
